# Efficacy and Safety of Renin-Angiotensin Aldosterone System Inhibitor in Patients with IgA Nephropathy: A Meta-Analysis of Randomized Controlled Trials

**Published:** 2019-09

**Authors:** Yu ZHAO, Heng FAN, Bei-Yan BAO

**Affiliations:** 1. Department of Nephrology, Ningbo Urology and Nephrology Hospital, Ningbo, China; 2. Department of Intensive Care Unit, Ningbo First Hospital, Ningbo, China

**Keywords:** Angiotensin converting enzyme inhibitor, Angiotensin receptor blocker, IgA nephropathy, Meta-analysis

## Abstract

**Background:**

Angiotensin converting enzyme inhibitor (ACEI) and angiotensin receptor blocker (ARB) as the commonly used renin-angiotensin aldosterone system inhibitor are widely used in patients with IgA nephropathy (IgAN), but the effect is controversy. In this study, we used a meta-analysis to evaluate the efficacy and safety of ACEI and/or ARB for the patients with IgAN.

**Methods:**

Two investigators independently searched the PubMed, EMBASE, the Cochrane Library, EBSCO, and Wiley databases without language restrictions. We collected the clinical randomized controlled trials (RCTs) on “ACEI and/or ARB for the patients with IgAN” published before December 31, 2018, and performed data extraction and quality analysis on the included studies, and analyzed data using RevMan 5.2 software.

**Results:**

A total of 10 RCTs (635 patients) were included in our analysis. Alone use of ACEI (MD=–0.75, 95%CI: –1.28–0.21, *P*=0.006) or ARB (MD=–0.56, 95%CI: –0.82–0.30, *P*< 0.001) or a combination of ACEI and ARB (MD=–0.63, 95%CI: –0.87–0.38, *P*<0.001) significantly reduced the levels of proteinuria in patients with IgAN. However, whether using ACEI or ARB alone or in combination with ACEI and ARB, there was no significant effect on serum creatinine, 24-creatinine clearance and glomerular filtration rate in patients with IgAN.

**Conclusion:**

The use of ACEI and ARB significantly reduces the levels of proteinuria in patients with IgAN, but more large-sample RCTs with long-term follow-up are needed for confirming our results and guiding clinical treatment.

## Introduction

IgA nephropathy (IgAN) is a glomerulonephritis characterized by diffuse deposition of IgA or IgA-based immunoglobulins in the mesangial membrane, which is the most common chronic glomerulonephritis in Asian (1). More and more studies have confirmed that it is not a benign disease with a good prognosis, but a progressive disease (2–4). The 10-year renal survival rate of IgAN patients ranges from 76% to 94%, and the 20-year renal survival rate ranges from 47% to 83% (5). About 30% of IgAN patients develop renal failure 20–30 years after the disease, and 1%–2% of the total number of patients diagnosed each year will enter end-stage renal failure (6).

A large number of proteinuria, recurrent episodes of gross hematuria, increased basal serum creatinine (Scr) and severe histopathological damage are risk factors of poor prognosis in IgAN patients (7, 8). Due to the variety of clinical and pathological types, there is still no uniform treatment for IgAN patients. There are also many controversial treatment options in clinical, including the treatment of hormones, the application of various immunosuppressive agents, and the control of blood pressure by angiotensin converting enzyme inhibitor (ACEI) or angiotensin receptor blocker (ARB).

Proteinuria is an independent risk factor for the prognosis of IgAN patients, and ACEI and ARB are often used to treat proteinuria, but the exact efficacy is still not fully understood (9). Especially for IgAN patients with proteinuria>l.0g/24 h proteinuria, whether the application of ACEI or ARB alone, or a combination of ACEI and ARB, and the efficacy of their applications are controversial.

Therefore, in this study, to evaluate the efficacy and safety of RASI for IgAN patients, we conducted a meta-analysis of clinical randomized controlled trials (RCTs) on RASI treatment of IgAN patients in recent years.

## Methods

### Search Strategy

Two investigators (YZ and HF) independently searched the Pubmed, Embase, the Cochrane Library, EBSCO, and Wiley databases, and collected the literatures on “RASI (ACEI and/or ARB) for the patients with IgAN” published before Dec 31, 2018 without language restrictions. We searched for different combinations of the following keywords, such as “ACEI” or “ARB” or “IgA nephropathy” or “IgAN” and “Proteinuria”. Inclusion criteria: 1) must be a clinical RCT; 2) complete data in the literature. Exclusion criteria: 1) not a clinical RCT; 2) patients treated with glucocorticoids and/or immunosuppressive agents; 3) RASI as an adjunct agents to clinical RCTs; 4) no clear instructions for missing patients during follow-up or no detailed description and analysis of results.

### Data Abstraction

We first browsed through all the retrieved literatures, removed duplicates, and excluded literatures that did not meet the inclusion criteria based on the title and abstract. Then we read the full text of the remaining literatures, and if the information provided in the RCTs was not comprehensive or in doubt, we would contact the author of the literature by E-mail to finalize the literature that was included in the analysis. Two investigators (YZ and HF) independently extracted the data and cross-checked. In case of disagreement, the third investigator (BYB) assisted in the judgment. Specific data included the title, authors, date of publication, source of the literature; number of patients, age, basic characteristics, treatment measures, follow-up time; changes in serum creatinine (Scr), 24-hour urine protein quantitation, 24 hours-creatinine clearance (24h-CrCl), glomerular filtration rate (GFR), adverse reactions, etc.

### Risk of Bias Assessment

According to the Cochrane bias risk method, the quality of the included studies were evaluated, including: the bias generated by the random sequence, the allocation of hidden bias, whether blind bias was applied to the patients and therapists, whether the bias of the complete prognosis was generated, whether the researchers selectively report bias, other unclear risk biases, etc.

### Statistical Analysis

We used RevMan 5.20 software for statistical analysis of the data. We used a χ^2^ test for heterogeneity analysis of the included studies, and the test level was *P*=0.10 and *I*^2^=50%. If *P*>0.10, *I*^2^<50% indicated that the heterogeneity between the results were low, and the fixed effect model was used for analysis; If *P*<0.10, *I*^2^>50% indicated that the heterogeneity between the results were high, and the random effects model was used for analysis. We used the mean difference (MD) as the effect variable of the continuous variable, and the results were represented by forest plots. We made a preliminary assessment of the publication bias by drawing a funnel plot and observing its symmetry, and then further evaluating the publication bias of the literature by Egger’s Test and Begg’s Test calculations. A *P*<0.05 indicates difference statistically significant.

## Results

### Literature collection

**Fig. 1:**
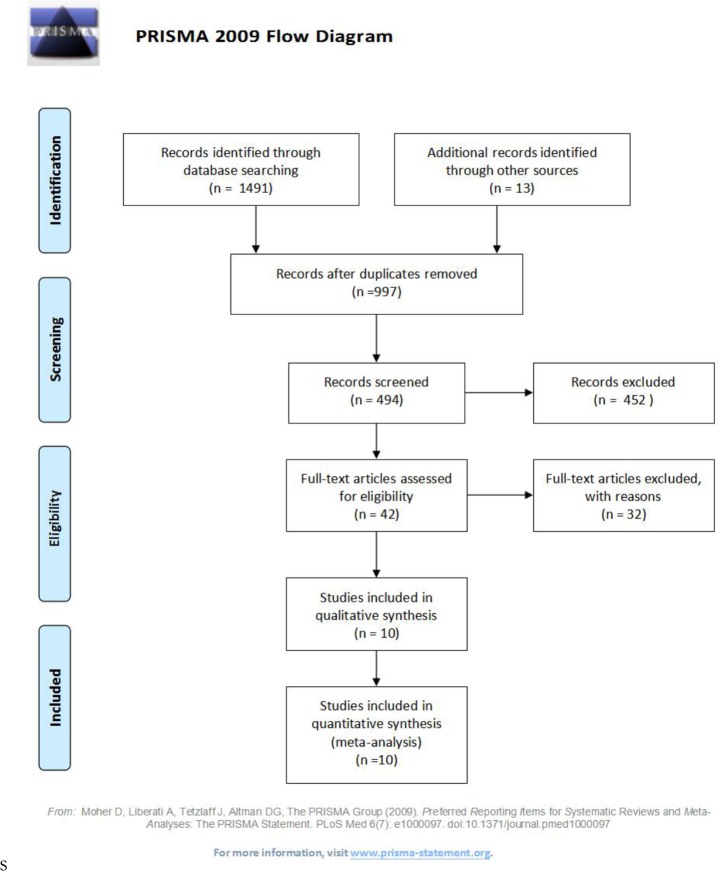
Study flow diagram

According to the standards we set, a total of 1,504 RCTs were retrieved, of which 1,173 were from PubMed, 142 were from EMBASE, 135 were from the Cochrane Library, 42 were from EBSCO, and 12 were from Wiley. After excluding duplicates, 997 articles were collected. Excluding 452 unrelated articles such as retrospective studies, basic research, other interventions, and non-RCT studies by reading topics and abstracts, the full text of the remaining 42 articles were reviewed. 32 articles were excluded from the relevant literature, due to no relevant data and/or RASI was not the only intervention drug in these studies. Finally, 10 RCTs (635 patients) were included in our analysis (Fig. 1) (9–18).

### Characteristics of trials

The basic characteristics of the included RCTs are detailed in Tables 1 and 2. Overall, 635 patients were enrolled in 10 RCTs, including 327 in the treatment group and 308 in the control group/placebo, and the follow-up period ranged from 1 to 276 months. 8 of these studies were from Asia (9–11, 13, 14, 16–18), 2 from Europe (12, 15), and 5 studies included children and minors (age < 18 years) (9, 12, 15, 16, 18). After further analysis of the RCTs, we found that 2 studies were ACEI+ARB compared with control group/placebo (9, 13), 5 studies were ACEI compared with control group/placebo (10–12, 15, 17), and 4 studies were ARB compared with control group/placebo (10, 14, 16, 18).

**Table 1:** Characteristics of randomized controlled trials involved in the studies

***Study***	***Country***	***No. of patients***	***Sex(Female /male)***	***Age(yr)***	***Baseline characteristics***	***Duration***	***Side effect***
Woo et al. (2000) (9)	Singapore	41	27/14	6 to 59	Proteinuria>1.0g/d, SCr> 1.4 mg/dL	December 1997 to December 1999	Not report
Nakamura et al. (2000) (10)	Japan	52	30/22	18 to 54	Proteinuria 1.0–3.0 g/d, GFR >80 ml/min/1.73m^2^	NA	Not report
Shiet al. (2002) (11)	China	131	44/87	18 to 42	Proteinuria>0.5g/d, Scr<5.9mg/dL	NA	Cough, Hypotension
Praga et al. (2003) (12)	Spain	44	17/27	16 to 43	Proteinuria>0.5 g/d, SCr< 1.5 mg/dL	September 1990 to September 1995	Hyperkalemia
Kim et al. (2003) (13)	Korea	19	9/10	24 to 38	Proteinuria>1.0g/d, GFR > 25–90 ml/min/1.73m^2^	NA	Azotemia, Hyperkalemia, Hypotension
Li et al. (2006) (14)	China	109	79/30	26 to 54	Proteinuria>1.0g/d, SCr<2.8mg/dL	June 2000 to June 2003	Allergy, Angioedema, Heart failure
Coppo et al. (2007) (15)	Italy, France, Portugal, Germany and Sweden	66	18/48	2 to 34	Proteinuria 1.0–3.5 g/d, GFR > 50 ml/min/1.73m^2^	November 1998 to December 2003	Cough
Shimizu et al. (2008) (16)	Japan	36	19/17	17 to 57	Proteinuria>0.4g/d g/d, eGFR > 50 ml/min/1.73m^2^	NA	Not report
Li et al. (2013) (17)	China	60	47/13	23 to 58	Proteinuria>0.5 g/d, SCr< 2.0 mg/dL	February 2004 to September 2005	Cough, Dizziness
Kohagura et al. (2018) (18)	Japan	77	43/34	15 to 70	Proteinuria>0.5 g/d, SCr< 1.5 mg/dl	April 2007 to December 2011	Worsen of hemoglobin A1c and partial alopecia

GFR, glomerular filtration rate; NA, not applicable; eGFR, estimated glomerular filtration rate

**Table 2:** Final outcomes of randomized controlled trials involved in the studies

***Study***	***No. of patients***		***Interventions***		***Follow-up (months)***	***Outcomes***	***Conclusions***
	***Treatment group***	***Control group***	***Treatment group***	***Control group***			
Woo et al. (2000) (9)	21	20	Enalapril+ Losartan	Control group	96–30	Scr, Proteinuria	Decreased Scr and urinary protein
Nakamura et al. (2000) (10)	32	20	Verapamil, Trandolapril, Candesartan	Placebo	3	Scr, Proteinuria, 24h-CrCl	Decreased urinary protein
Shi et al. (2002) (11)	65	66	Benazepril	Control group	1–276	Scr, Proteinuria, 24h-CrCl	Decreased urinary protein
Praga et al. (2003) (12)	23	21	Enalapril	Control group	29–120	Scr, Proteinuria, 24h-CrCl	Decreased Scr and urinary protein
Kim et al. (2003) (13)	12	7	Candesartan+ Ramipril	Placebo	>6	Scr, Proteinuria, 24h-CrCl	Decreased urinary protein
Li et al. (2006) (14)	54	55	Valsartan	Placebo	24	Scr, Proteinuria, GFR	Decreased urinary protein and Improve renal function
Coppo et al. (2007) (15)	32	34	Benazepril	Placebo	Up to 58	Proteinuria, GFR, 24h-CrCl	Decreased urinary protein and Improve renal function
Shimizu et al. (2008) (16)	18	18	Losartan	Control group	12	Scr, Proteinuria, GFR	Decreased urinary protein
Li et al. (2013) (17)	30	30	Ramipril	Control group	60	Scr, Proteinuria, GFR	No benefit
Kohagura et al. (2018) (18)	40	37	Candesartan	Control group	24	Scr, Proteinuria, GFR	Decreased urinary protein

Scr, Serum creatinine; BUN, blood urea nitrogen; 24h-CrCl, 24 hours-creatinine clearance; GFR, glomerular filtration rate;

### Quality evaluation

**Fig. 2:**
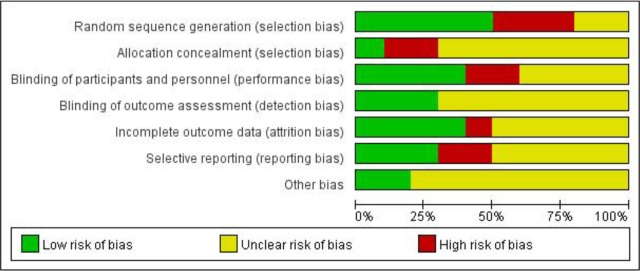
Risk of bias graph

**Fig. 3:**
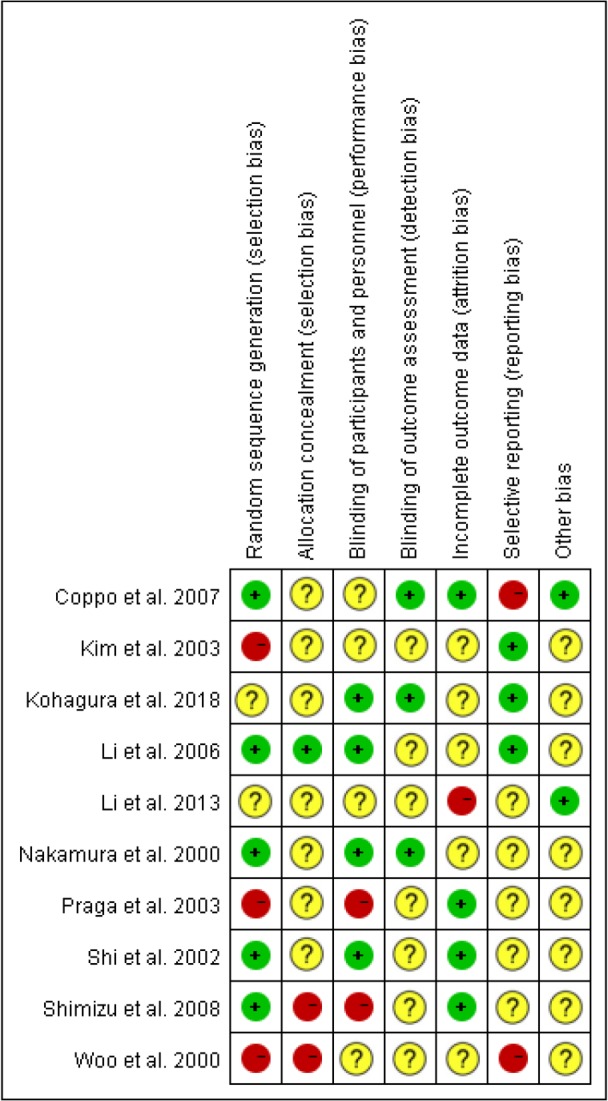
Risk of bias summary. “+”low risk of bias, “−”high risk of bias, “?”unclear risk of bias

5 RCTs referred to random grouping (9, 11, 14–16), but only 2 RCTs specifically described the random grouping method applied (14, 15). Only 1 RCT was double-blind (14), and the rest of the study only blinded the results. The results of all study data were complete, and selective reporting and other bias were not significant (Figs. 2, 3).

### Clinical outcomes

**Fig. 4:**
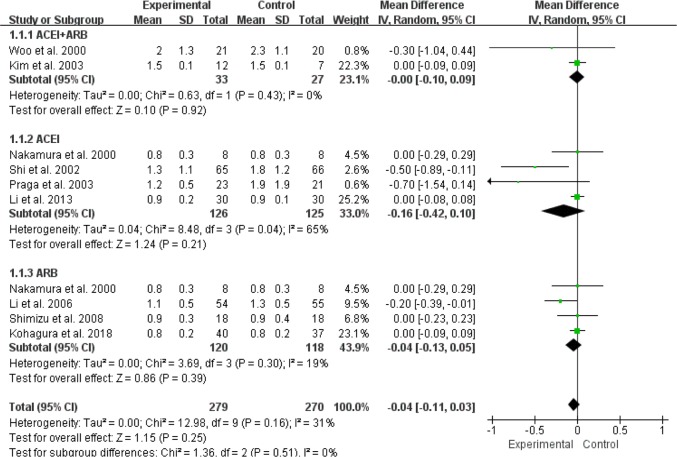
RASI for decreasing the level of Scr in patients with IgA nephropathy. RASI, renin-angiotensin aldosterone system inhibitor; Scr, serum creatinine; ACEI, angiotensin-converting enzyme inhibitor; ARB, angiotensin-receptor blocker

**Fig. 5:**
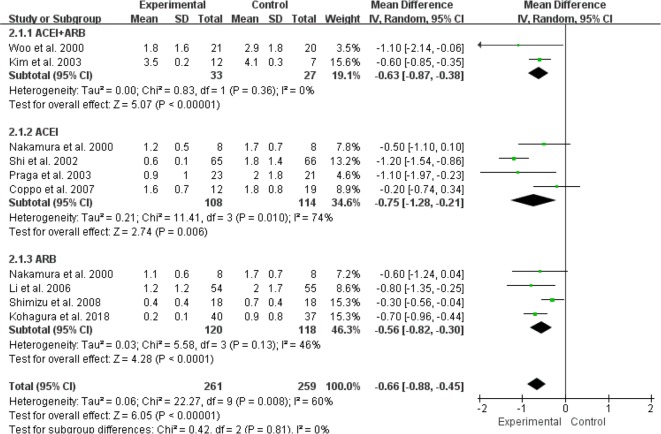
RASI for decreasing proteinuria in patients with IgA nephropathy. RASI, renin-angiotensin aldosterone system inhibitor; ACEI, angiotensin-converting enzyme inhibitor; ARB, angiotensin-receptor blocker

For the effect of RASI on Scr levels in patients with IgAN, 2 RCTs (60 patients) were ACEI+ARB compared with control group/placebo (9, 13), 4 studies (287 patients) were ACEI compared with control group/placebo (10–12, 17), and 4 studies (274 patients) were ARB compared with control group/placebo (10, 14, 16, 18). We used a random effects model to analyze the results, and our results suggested that either ACEI (MD=–0.16, 95% CI: –0.42–0.10, *P*=0.21) or ARB (MD=–0.04, 95% CI: –0.13–0.05, *P*=0.39) alone, or a combination of ACEI and ARB (MD=–0.00, 95%CI:–0.10–0.09, *P*=0.92), had no effect on changes in Scr levels (Fig. 4). In terms of the effect of RASI on proteinuria levels in IgAN patients, 2 RCTs (60 patients) were ACEI+ARB compared with control group/placebo (9, 13), and 4 studies (353 patients) were ACEI compared with control group/placebo (10–12, 15, 17), 4 studies (274 patients) were ARB compared with control group/placebo (10, 14, 16, 18). We used a random effects model analysis and the results suggested that alone use of ACEI (MD=–0.75, 95%CI: –1.28–0.21, *P*=0.006) or ARB (MD=–0.56, 95%CI: –0.82–0.30, *P*< 0.001) or a combination of ACEI and ARB (MD=–0.63, 95%CI: –0.87–0.38, *P*<0.001) significantly reduced the levels of proteinuria in patients with IgAN (Fig. 5).

**Fig. 6:**
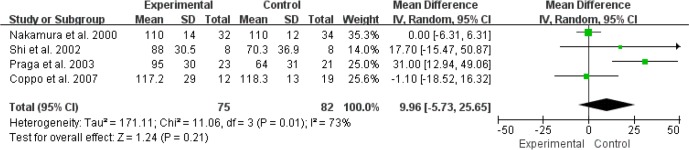
ACEI for improving24h-CrCl in patients with IgA nephropathy. ACEI, angiotensin-converting enzyme inhibitor; 24h-CrCl, 24hours-creatinine clearance

**Fig. 7:**
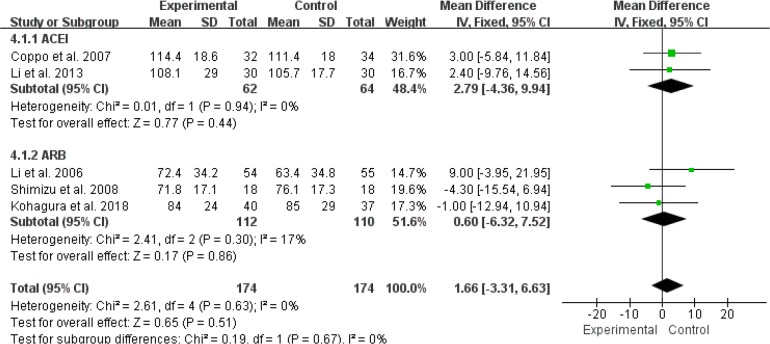
RASI for improving the GFR in patients with IgA nephropathy. RASI, renin-angiotensin aldosterone system inhibitor; GFR, glomerular filtration rate; ACEI, angiotensin-converting enzyme inhibitor; ARB, angiotensin-receptor blocker

For the effect of RASI on 24h-CrCl in patients with IgAN, 4 RCTs (276 patients) were ACEI compared with control group/placebo (10–12, 15). We used a random effects model to analyze the results, and our results suggested that ACEI (MD=9.96, 95% CI: –5.73–25.65, P= 0.21) had no significant effect on 24h-CrCl in IgAN patients (Fig. 6). In terms of the efficacy of RASI on GFR in IgAN patients, 2 RCTs (126 patients) were ACEI compared with control group/placebo (15, 17), and 3 studies (222 patients) were ARB compared with control group/placebo (14, 16, 18). We used a random effects model analysis and the results suggested that the alone use of either ACEI (MD=2.79, 95%CI: –4.36–9.94, *P*=0.44) or ARB (MD=0.60, 95%CI:–6.32–7.52, *P*=0.86) had no significant therapeutic effect on GFR in patients with IgAN (Fig. 7).

### Publishing bias

**Fig. 8:**
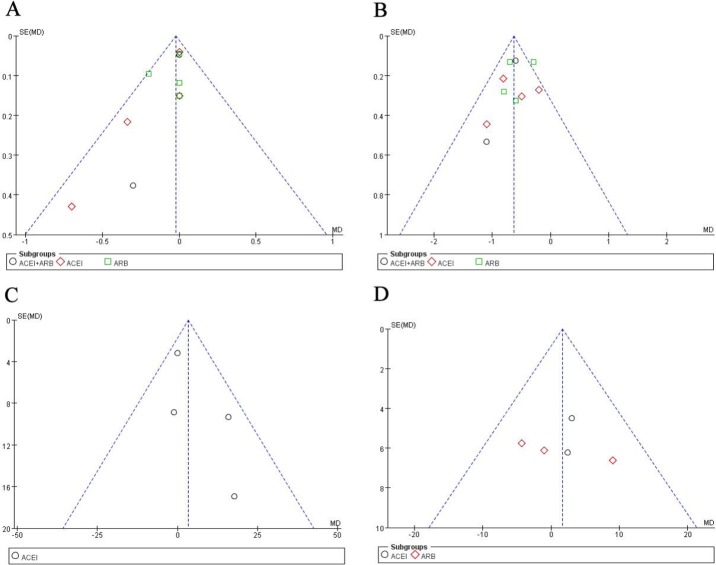
Funnel plots of meta-analysis. The levels of serum creatinine (A, Begg’s test, *P*=0.31; Egger’s test, *P*=0.42), the levels of proteinuria (B, Begg’s test, *P*=0.12; Egger’s test, *P*=0.64),24 hours-creatinine clearance (C, Begg’s test, *P*=0.53; Egger’s test, *P*=0.63), and the glomerular filtration rate(D, Begg’s test, *P*=0.21; Egger’s test, *P*=0.48). The results revealed no publication bias, as all *P* values were>0.05. ACEI, angiotensin-converting enzyme inhibitor; ARB, angiotensin-receptor blocker; SE, standard error; MD, mean difference

We analyzed the results of different literatures in the subgroup. Our results showed that the scatters of each literature were symmetrically distributed on both sides of the line in different gradient analysis. The large samples were at the end and the small samples were at the top. All literature analyzed did not have significant publication bias (Fig. 8).

## Discussion

In clinical, IgAN patients with high blood pressure require strict blood pressure control. If there is no contraindication, patients are generally recommended to use ACEI and/or ARB to control blood pressure within the ideal range (19). Many studies indicated that ACEI/ARB can benefit IgAN, which is reflected in its dual effects of lowering blood pressure and kidney protection (9–12). However, nearly 20% of IgAN patients still would be progress to end-stage renal disease after receiving ACEI (20). Long-term use of ACEI will cause angiotensin converting enzyme escape, while ARB may release renin and vascular tone through negative feedback mechanism of renin-angiotensin aldosterone system (RAS) compensating for ACEI defects, so more studies are beginning to investigate the combination of ACEI and ARB (21).

In this study, we included 10 RCTs, 5 of which used ACEI, 4 of which used ARB, and 2 of which used ACEI+ARB. We first explored the effect of RASI on Scr levels in patients with IgAN. Our results showed that neither ACEI nor ARB alone or in combination of ACEI and ARB had significant effects on Scr levels in IgAN patients. Therefore, it is indicated that the therapeutic effect of RASI on IgAN patients with different renal functions are not significant. Some studies had also pointed out that ACEI combined with hormones can effectively improve renal function compared with the simple application of ACEI, but still need multi-center and large sample clinical RCTs to demonstrate (22–24).

Proteinuria is not only one of the manifestations of renal lesions, but also an independent factor of renal damage, which is positively correlated with the severity of renal disease (25). Therefore, proteinuria is an important factor in determining the prognosis of IgAN patients. For glomerular disease with proteinuria, due to the RAS activation state, blood pressure should be strictly controlled (26). For IgAN patients with small amount of proteinuria, the blood pressure target should be controlled <130/80mmHg, and for patients with large amounts of protein, the blood pressure should be controlled more strictly <125/75mmHg (27). Therefore, we used a proteinuria index for comprehensive analysis to evaluate the efficacy of RASI in patients with IgAN. The results showed that the use of either ACEI or ARB alone or the combination of ACEI and ARB was superior to the control group in reducing proteinuria, suggesting that the use of ACEI and ARB can significantly reduce proteinuria in patients with IgAN.

Recently, Ji Y et al (28) reported their latest meta-analysis results. By comparing the effect of ACEI/ARB and control group on proteinuria in IgAN patients, it was found that the use of ACEI/ARB can significantly reduce proteinuria. But they did not compare ACEI and ARB separately, which made their results unreliable. However, we compared the effect of ACEI and/or ARB on proteinuria in patients with IgAN separately, and the results were more accurate and informative. Further, we also analyzed the effects of RASI on 24h-CrCl and GFR in patients with IgAN. Our results suggested that the use of RASI had no effect on 24h-CrCl and GFR in patients with IgAN, which was consistent with previous results (29).

Low-dose RASI may have poor effect, while high-dose may bring more side effects. Therefore, the dose of RASI has always been one of the concerns of clinicians. Our results showed that different RASI had different therapeutic doses, and the specific dose was mainly determined by the patient’s blood pressure and renal function. Among the results of our analysis, 7 RCTs mentioned adverse events of RASI, and common adverse events include: cough, hypotension, hyperkalemia, allergy and dizziness. It was suggested that we could adjust the dose of RASI appropriately in consideration of the side effects of the drugs under the same effect.

Our meta-analysis has the following limitations. First, we did not limit the age of patients, and 5 of the RCTs included children, minors and adults, and it was difficult to quantify them by age through analysis. Second, the follow-up time of the included studies was different, range from 1 to 276 months, the effect of RASI on IgAN patients may vary at different periods. Third, the types and doses of ACEI and/or ARB used in the study were different, and it was difficult to unify them by calculation. Fourth, our study did not analyze adverse events, because the different adverse events mentioned in the RCTs, and it was difficult to analyze the adverse events of these studies by meta-analysis.

## Conclusion

The RCTs we included in the analysis are small sample studies and still lack relevant large sample clinical RCTs. Alone use of ACEI or ARB or a combination of ACEI and ARB significantly reduced the levels of proteinuria in patients with IgAN. The treatment effect of single drug was poor or proteinuria >1.0g/24 h, which might be an indication for combination therapy. However, there is no conclusion on the protective effect of renal function and the delay in the progression of renal failure, suggesting that further multi-center, large-sample clinical RCTs with long-term follow-up are needed to obtain more accurate and effective information for guiding clinical treatment.

## Ethical considerations

Ethical issues (Including plagiarism, informed consent, misconduct, data fabrication and/or falsification, double publication and/or submission, redundancy, etc.) have been completely observed by the authors.

## References

[1] Li G Wu W Zhang X (2018). Serum levels of tumor necrosis factor alpha in patients with IgA nephropathy are closely associated with disease severity. BMC Nephrol, 19(1):326. 30428849 10.1186/s12882-018-1069-0 PMC6236996

[2] Selvaskandan H Cheung CK Muto M Barratt J (2019). New strategies and perspectives on managing IgA nephropathy. Clin Exp Nephrol, 23(5):577–88. 30756248 10.1007/s10157-019-01700-1 PMC6469670

[3] Andeen NK Jefferson JA Akilesh S (2018). IgA-dominant glomerulonephritis with a membranoproliferative pattern of injury. Hum Pathol, 81:272–80. 30420049 10.1016/j.humpath.2018.06.031

[4] Zhang JJ Yu GZ Zheng ZH (2017). Dividing CKD stage 3 into G3a and G3b could better predict the prognosis of IgA nephropathy. PLoS One, 12(4):e0175828. 28414748 10.1371/journal.pone.0175828 PMC5393865

[5] Oshima Y Moriyama T Itabashi M (2015). Characteristics of IgA nephropathy in advanced-age patients. Int Urol Nephrol, 47(1):137–45. 25388352 10.1007/s11255-014-0872-1

[6] Imai N Shirai S Yasuda T (2016). Long-term prognosis of IgA nephropathy presenting with minimal or no proteinuria: A single center experience. Indian J Nephrol, 26(2):107–12. 27051134 10.4103/0971-4065.157010 PMC4795425

[7] Park S Baek CH Park SK (2019). Clinical Significance of Crescent Formation in IgA Nephropathy - a Multicenter Validation Study. Kidney Blood Press Res, 44(1):22–32. 30808840 10.1159/000497808

[8] Liu D You J Liu Y (2019). Serum immunoglobulin G provides early risk prediction in immunoglobulin A nephropathy. Int Immunopharmacol, 66:13–8. 30415190 10.1016/j.intimp.2018.10.044

[9] Woo KT Lau YK Wong KS Chiang GS (2000). ACEI/ATRA therapy decreases proteinuria by improving glomerular permselectivity in IgA nephritis. Kidney Int, 58(6):2485–91. 11115082 10.1046/j.1523-1755.2000.00432.x

[10] Nakamura T Ushiyama C Suzuki S (2000). Effects of angiotensin-converting enzyme inhibitor, angiotensin II receptor antagonist and calcium antagonist on urinary podocytes in patients with IgA nephropathy. Am J Nephrol, 20(5):373–9. 11092994 10.1159/000013619

[11] Shi X Chen X Liu S (2002). The effects of angiotensin-converting enzyme inhibitor on IgA nephropathy and the influencing factors. Zhonghua Nei Ke Za Zhi, 41(6):399–403. (Article in Chinese) 12137603

[12] Praga M Gutiérrez E González E (2003). Treatment of IgA nephropathy with ACE inhibitors: a randomized and controlled trial. J Am Soc Nephrol, 14(6):1578–83. 12761258 10.1097/01.asn.0000068460.37369.dc

[13] Kim MJ Song JH Suh JH (2003). Additive antiproteinuric effect of combination therapy with ACE inhibitor and angiotensin II receptor antagonist: differential short-term response between IgA nephropathy and diabetic nephropathy. Yonsei Med J, 44(3):463–72. 12833584 10.3349/ymj.2003.44.3.463

[14] Li PK Leung CB Chow KM (2006). Hong Kong study using valsartan in IgA nephropathy (HKVIN): a double-blind, randomized, placebo-controlled study. Am J Kidney Dis, 47(5):751–60. 16632013 10.1053/j.ajkd.2006.01.017

[15] Coppo R Peruzzi L Amore A (2007). IgACE: a placebo-controlled, randomized trial of angiotensin-converting enzyme inhibitors in children and young people with IgA nephropathy and moderate proteinuria. J Am Soc Nephrol, 18(6):1880–8. 17513327 10.1681/ASN.2006040347

[16] Shimizu A Takei T Uchida K (2008). Low-dose losartan therapy reduces proteinuria in normotensive patients with immunoglobulin A nephropathy. Hypertens Res, 31(9):1711–7. 18971549 10.1291/hypres.31.1711

[17] Li PK Kwan BC Chow KM (2013). Treatment of early immunoglobulin A nephropathy by angiotensin-converting enzyme inhibitor. Am J Med, 126(2):162–8. 23331443 10.1016/j.amjmed.2012.06.028

[18] Kohagura K Arima H Miyasato H (2018). Add-On Effect of Angiotensin Receptor Blockade (Candesartan) on Clinical Remission in Active IgA Nephropathy Patients Treated with Steroid Pulse Therapy and Tonsillectomy: a Randomized, Parallel-Group Comparison Trial. Kidney Blood Press Res, 43(3):780–92. 29794482 10.1159/000489914 PMC6019550

[19] Coppo R (2018). Treatment of IgA nephropathy: Recent advances and prospects. Nephrol Ther, 14 Suppl 1:S13–S21. 29606258 10.1016/j.nephro.2018.02.010

[20] Shima Y Nakanishi K Sako M (2019). Lisinopril versus lisinopril and losartan for mild childhood IgA nephropathy: a randomized controlled trial (JSKDC01 study). Pediatr Nephrol, 34(5):837–46. 30284023 10.1007/s00467-018-4099-8

[21] Nakamura T Inoue T Sugaya T (2007). Beneficial effects of olmesartan and temocapril on urinary liver-type fatty acid-binding protein levels in normotensive patients with immunoglobin A nephropathy. Am J Hypertens, 20(11):1195–201. 17954367 10.1016/j.amjhyper.2007.06.003

[22] Tam FWK Pusey CD (2018). TESTING corticosteroidsin IgA nephropathy: a continuing challenge. Clin J Am Soc Nephrol, 13(1):158–160. 29237704 10.2215/CJN.10560917 PMC5753322

[23] Lv J Zhang H Wong MG (2017). Effect of oral methylprednisolone on clinical outcomes in patients with IgA nephropathy: the TESTING Randomized Clinical Trial. JAMA, 318 (5):432–42. 28763548 10.1001/jama.2017.9362 PMC5817603

[24] Qian G Zhang X Xu W (2019). Efficacy and safety of glucocorticoids for patients with IgA nephropathy: a meta-analysis. Int Urol Nephrol, 51(5):859–68. 30843135 10.1007/s11255-019-02094-5

[25] Glassock RJ (2019). Mortality Risk in IgA Nephropathy. J Am Soc Nephrol, 30(5):720–2. 31040186 10.1681/ASN.2018121255 PMC6493985

[26] Horita Y Taura K Taguchi T (2006). Aldosterone breakthrough during therapy with angiotensin-converting enzyme inhibitors and angiotensin II receptor blockers in proteinuric patients with immunoglobulin A nephropathy. Nephrology (Carlton), 11(5):462–6. 17014562 10.1111/j.1440-1797.2006.00665.x

[27] Pozzi C Del Vecchio L Casartelli D (2006). ACE inhibitors and angiotensin II receptor blockers in IgA nephropathy with mild proteinuria: the ACEARB study. J Nephrol, 19(4):508–14. 17048209

[28] Ji Y Yang K Xiao B (2019). Efficacy and safety of angiotensin-converting enzyme inhibitors/angiotensin receptor blocker therapy for IgA nephropathy: A meta-analysis of randomized controlled trials. J Cell Biochem, 120(3):3689–95. 30270542 10.1002/jcb.27648

[29] Cheng J Zhang X Tian J (2012). Combination therapy an ACE inhibitor and an angiotensin receptor blocker for IgA nephropathy: a meta-analysis. Int J Clin Pract, 66(10):917–23. 22994326 10.1111/j.1742-1241.2012.02970.x

